# Risk Factors for Malignancy of Thyroid Nodules in Patients Undergoing Thyroid Resection

**DOI:** 10.3390/jcm13247559

**Published:** 2024-12-12

**Authors:** Anna Krzentowska, Filip Gołkowski, Elżbieta Broniatowska, Aleksander Konturek, Marcin Barczyński

**Affiliations:** 1 Department of Endocrinology and Internal Medicine, Medical College, Andrzej Frycz Modrzewski Krakow University, 30-705 Kraków, Poland; fgolkowski@afmmed.edu.pl; 2 Faculty of Medicine and Health Sciences, Medical College, Andrzej Frycz Modrzewski Krakow University, 30-705 Kraków, Poland; ebroniatowska@afm.edu.pl; 3 Department of Endocrine Surgery, Faculty of Medicine, Jagiellonian University Medical College, 31-501 Kraków, Poland; aleksander.konturek@uj.edu.pl (A.K.); marcin.barczynski@uj.edu.pl (M.B.)

**Keywords:** cytopathological correlation, fine-needle aspiration, Bethesda category, thyroid cancer incidence

## Abstract

**Background:** An accurate diagnosis of thyroid nodules is crucial for avoiding unnecessary surgical procedures and making timely treatment possible. The objective of the present study was to evaluate the diagnostic accuracy of fine-needle aspiration biopsy (FNAB) using histopathological findings as the reference standard. Patients with the diagnostic categories (DCs) III, IV, and V were subjected to special analysis. In addition, the authors assessed whether other factors, including age, gender, body mass index (BMI), obesity, and histopathologically confirmed lymphocytic thyroiditis, had an impact on the occurrence of malignant tumors. **Methods**: We performed a retrospective analysis of 535 patients (with a mean age of 52.3) who underwent thyroid surgery between October 2022 and September 2023 at the Department of Endocrine Surgery at the University Hospital in Krakow. To assess the reliability of FNAB, the results obtained using the Bethesda classification were compared with the histopathological results. **Results:** The risk of malignancy (ROM) values for DCs I–VI were 38.1%, 15.6%, 29.8%, 18.6%, 91.0%, and 93.2%, respectively. DC V (OR 62.34, *p* < 0.0001) and an age ≤ 50 (OR = 2.31, *p* < 0.006) had statistically significant effects on the risk of thyroid cancer. DCs III and IV were not statistically significantly associated with the risk of malignancy (OR = 1.68, *p* = 0.16; OR = 1.51, *p* = 0.3, respectively). There were no statistically significant differences in sex, BMI, or obesity between the patients with benign and malignant lesions. **Conclusions**: DC V is associated with a high likelihood of malignancy, especially in patients under 50 years of age, and, therefore, surgery is indicated in this category of subjects. In DCs III and IV, the risk of malignancy is lower, and conservative management with active clinical and ultrasound surveillance can be considered. In patients < 50 years of age, with Bethesda categories III and IV, surgical treatment should be considered.

## 1. Introduction

Thyroid nodules are common and usually asymptomatic, with a prevalence ranging between 26 and 67%, according to the literature [1,2,3]. The majority of these nodules are benign; nevertheless, approximately 8% of them are malignant [4]. Recently, the worldwide incidence of thyroid cancer has increased remarkably [5]. Therefore, the correct diagnosis of thyroid nodules is essential to avoid unnecessary surgery and to allow for the prompt treatment of malignant tumors. Ultrasound (US) is the primary imaging test for the diagnosis of thyroid cancer and other thyroid diseases [6,7]. According to the 5-stage EUTIRADS classification, the risk of malignancy depends on the characteristics of the focal lesion and is close to 0% for categories I and II, 2–4% for category III, 6–17% for category IV, and more than 26–87% for category V [8]. Fine-needle aspiration biopsy (FNAB) is a good diagnostic tool for thyroid nodules; however, the high false negative rate of results obtained using this method renders it controversial.

In 2007, the Bethesda System for the Reporting of Thyroid Cytopathology (TBSRTC) was introduced, and the risk of malignancy for the six diagnostic categories (DCs) was defined. For DCs I, II, III, IV, V, and VI, the percentages were 1–4%, 0–3%, 5–15%, 15–30%, 60–75%, and 97–99%, respectively [9]. 

Subsequently, the second edition of the TBSRTC was published, which showed a higher risk of malignancy for the lower Bethesda diagnostic categories. It is apparent that the biopsy results affect the extent of the surgical procedure [10,11,12].

Cytological diagnoses in Bethesda categories III and IV account for approximately 30% of FNAB results [13], and the management of this group of patients varies widely, from clinical and ultrasound follow-up to repeated FNAB, molecular testing, and thyroid surgery. In the case of Bethesda category V, patients are generally referred for surgery. 

The objective of the present study was to assess the risk of malignancy in patients referred for surgery for thyroid nodules, focusing on DCs III, IV, and V. In addition, an attempt was made to assess whether other factors, i.e., age, sex, BMI (body mass index), obesity, and coexisting lymphocytic thyroiditis, had an impact on increasing the risk of malignancy in thyroid nodules.

## 2. Materials and Methods

### 2.1. Patients and Methods

The authors performed a retrospective data analysis of 535 patients who underwent surgery at the Department of Endocrine Surgery of the University Hospital in Krakow between October 2022 and September 2023. Data were collected for all the patients referred for surgery due to a nodular goiter, suspected malignancy, or a finding of malignancy through FNA. Patients who were reoperated for metastatic or recurrent cancer in the lymph nodes were excluded from this study (14 cases). The entire group of patients (n = 521) was evaluated first, followed by an assessment of the patients divided into groups according to Bethesda DCs III, IV, and V (n = 221). The results of particular patients were anonymized and collected in the international EUROCRINE database [14]. To assess the reliability of FNAB, the results obtained according to the Bethesda classification were compared with the histopathological results. In addition, parameters such as age, sex, BMI, obesity, and the presence of lymphocytic thyroiditis in histopathological examination were analyzed. 

### 2.2. Diagnosis and Evaluation

Each patient underwent preoperative thyroid ultrasonography (US) and preoperative FNAB of the suspected focal lesions. The patients were referred from various outpatient clinics, so the EUTIRADS thyroid lesion assessment was not performed in every case. Therefore, this parameter was not included in the calculation. The thyroid US-guided FNAB procedure and the evaluation of the FNAB cytology slides were performed prior to admission to the hospital. The FNAB results were classified based on the criteria according to TBSRTC. The patients underwent total thyroidectomies or unilateral thyroid lobectomies. The extent of lymphadenectomy depended on the results of the preoperative FNAB and preoperative staging of the disease. The postoperative material was examined histopathologically. The histopathological diagnoses of the thyroid nodules were established according to the World Health Organization 2022 classification guidelines [15]. In addition, the histopathological examination assessed the presence of other pathological changes, such as lymphocytic thyroiditis, among others. 

The histopathological findings were divided into two groups: benign and malignant. The authors assessed which Bethesda categories featured benign nodules and which featured malignant nodules. The histopathological results of the Bethesda III, IV, and V patients were compared with the histopathological results of the Bethesda II patients using multivariate regression analysis. 

### 2.3. Statistical Analysis 

The continuous variables were presented as the means, standard deviations (SDs), medians, and quartiles (lower and upper). The Shapiro–Wilk test was used to verify the assumption of the normal distribution of the continuous variables. The Mann–Whitney U-test was performed to compare two groups because of the lack of normality for continuous variables. Due to the departures from normality, the Kruskal–Wallis test was applied for the comparison of the continuous variables between more than two groups. Post hoc analysis was also performed. The qualitative variables were presented as numbers (percentages), and the Chi-square test was used for comparing them between two or more groups. Receiver operating characteristic (ROC) curves were created to determine the Bethesda category manifesting malignancy. The multivariable logistic regression was used to determine the risk factors of malignancy. The results are shown as the odds ratios (ORs) with 95% confidence intervals (CI). The Hosmer–Lemehsow test was used as the fit of measure for the logistic regression model. The McFadden coefficient was also calculated; a value of 0.3 indicates good fitting. To check the prediction power of the logistic regression model, the c-statistics (i.e., the area under the curve (AUC)) was applied. An AUC value of 0.82 also indicates relatively high prediction power of the presented model. The outlier points were also verified for this model. *p*-values below 0.05 were considered statistically significant in the two-sided tests. Statistical analyses were conducted to calculate the sensitivity, specificity, positive predictive value (PPV), negative predictive value (NPV), false positive rate (FP), false negative rate (FN), true positive rate (TP), and true negative rate (TN). In addition, multivariate logistic regression was performed to assess the risk of malignancy (ROM).

The R v. 4.4.2 package [16] and Statistica 13 software (StatSoft Inc., Tulsa, OK, USA) were used to perform the analyses.

## 3. Results

### 3.1. Results Obtained from the Entire Study Group of Patients (n = 521)

The group of 521 patients included 96 men (18.4%) and 425 women (81.5%) (a ratio of 1:4.4), with a mean age of 52.3 ± 15.2 years. The reasons for referral to surgery were as follows: exclusions of malignant neoplasms (32.6%), the presence of malignancies (22.2%), compression symptoms (19.5%), thyrotoxicosis (8.6%), and other indications (18.2%). The demographic and pathologic characteristics of the entire group are shown in Table 1.

In this study population, 37.8% (197 cases) of the thyroid tumors were malignant. The classification of the types of malignant thyroid neoplasms (n = 197) by sex was as follows: PTC in 147 (74.65%) females and 25 (12.6%) males; FTC in 9 (4.5%) females and 7 (3.5%) males; MTC in 2 (1.0%) females and 3 (1.5%) males; ATC in 1 (0.5%) male; lymphoma in 1 (0.5%) female.

Among the 172 cases of PTC, the following subtypes were identified: classic PTC in 121 cases (70.4%); follicular variant of PTC in 44 cases (25.5%); encapsulated variant of PTC in 3 cases (1.8%); other unusual variants of PTC in 4 cases (2.3%). 

In 16 cases of FTC, the following subtypes were found: widely invasive FTC in 3 cases (18.7%); minimally invasive FTC in 8 cases (50.0%); encapsulated angioinvasive FTC in 4 cases (25.0%); no data in 1 case (6.2%). 

The following low-risk neoplasms were noted: follicular tumors with uncertain malignant potential (FT-UMP) in 8 cases (1.5%); non-invasive follicular thyroid neoplasms with papillary-like nuclear features (NIFTP) in 10 cases (1.8%); well-differentiated tumors of uncertain malignant potential (WDT-UMP) in 4 cases (0.7%). 

The cytological and histopathological results showed a statistically significant association (*p* < 0.0001) between age and the presence of thyroid malignancy. The patients with confirmed thyroid cancer were, on average, 8 years younger than the patients without malignancies. The mean age of the patients with malignant thyroid cancer was 47.4 ± 14.9 years (median of 45.0), while the mean age of the patients with a benign diagnosis was 55.3 ± 14.7 years (median of 57.0). Among the patients in DC V, malignancy was diagnosed in more than 90% of the cases. In contrast, in the DC III group, benign lesions were found in 59 cases (70.24%) and malignant lesions in 25 cases (29.76%). In DC IV, benign lesions were found in 57 cases (81.4%) and malignant lesions in 13 cases (18.6%). There were no statistically significant associations between gender (*p* = 0.76), BMI (*p* = 0.52), and obesity (*p* = 0.76) and the presence of thyroid malignancy.

In addition, the presence of lymphocytic thyroiditis (LT) was assessed histopathologically in the operated patients. LT was found in 79 patients (14.8%), i.e., in 47 patients (59.5%) with a diagnosis of malignant thyroid cancer and in 32 patients (40.5%) diagnosed with benign thyroid lesions. The following types of thyroid cancer were found in people with LT in the background: FTC in 2 (4.2%) females; OTC in 1 (2.1%) female; PTC in 44 (93.6%) patients, including 40 females (91.0%) and 4 males (9.0%).

The histopathological findings of the patients in the Bethesda DC I, II, III, IV, V, and VI groups are shown in Table 2.

The risk of malignancy (ROM) was calculated for each Bethesda category (Table 3).

**Table 3:** Risk of malignancy (ROM).

Cytology	No. of Malignant Cases (All)	ROM %
I	8 (21)	38.1
II	28 (180)	15.6
III	25 (84)	29.8
IV	13 (70)	18.6
V	61 (67)	91
VI	55 (59)	93.2

In order to assess the relevance of FNAB, the authors determined five groups of subjects (criteria 2–6) for which the following diagnostic values were analyzed: TP, TN, FP, FN, PPV, and NPV. We calculated the biopsy sensitivity according to the inclusion of different Bethesda categories in the numerator (Table 4).

**Table 4:** Changes in the sensitivity for malignancy according to the criteria for a positive diagnosis (excluding patients without cytology performed).

Criterion	TP	FP	FN	TN	Sensitivity	Specificity	PPV	NPV
**6**	55	4	135	287	0.29 (0.23, 0.36)	0.99 (0.97, 1.00)	0.93 (0.84, 0.98)	0.68 (0.63, 0.72)
**5**	116	10	74	281	0.61 (0.54, 0.68)	0.97 (0.94, 0.98)	0.92 (0.86, 0.96)	0.79 (0.75, 0.83)
**4**	129	67	61	224	0.68 (0.61, 0.74)	0.77 (0.72, 0.82)	0.66 (0.59, 0.72)	0.79 (0.73, 0.83)
**3**	154	126	36	165	0.81 (0.75, 0.86)	0.57 (0.51, 0.62)	0.55 (0.49, 0.61)	0.82 (0.76, 0.87)
**2**	182	278	8	13	0.96 (0.92, 0.98)	0.04 (0.02, 0.08)	0.40 (0.35, 0.44)	0.62 (0.38, 0.82)

Legend: The diagnostic categories (DC) assigned to the numerator and denominator for each criterion were as follows: criterion 6: DCs VI/V, IV, III, II, and I; criterion 5: DCs VI, V/IV, III, II, and I; criterion 4: DCs VI, V, IV/III, II, and I; criterion 3: DCs VI, V, IV, III/ II, and I; criterion 2: DCs VI, V, IV, III, and II/I. TP, true positive; TN, true negative; FP, false positive; FN, false negative; PPV, positive predictive value; NPV, negative predictive value.

Explanation: criterion 6 indicates a positive diagnosis only within the DC VI group relative to all other categories. Criterion 5 indicates a positive diagnosis within the DCs VI and V relative to all other categories. 

The highest positive predictive value (PPV) confirming malignancy through histopathological examination for criterion 6 was 0.93, and for criterion 5, it was 0.92. For the subsequent criteria, the PPVs were as follows: criterion 4—0.66; criterion 3—0.55; criterion 2—0.40.

**Figure 1:**
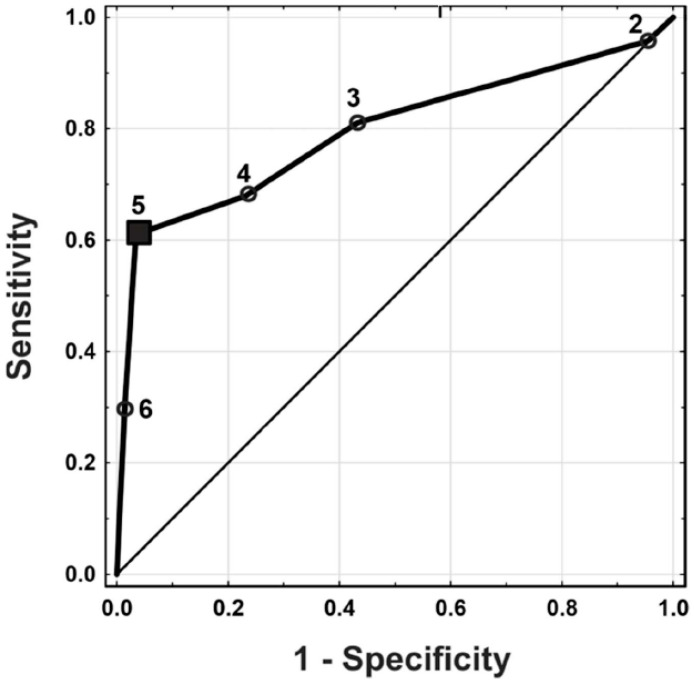
ROC curve of probability of malignancy for criteria 2–6.

An ROC curve was then determined, from which it was concluded that patients in DCs V and VI (criterion 5) would have thyroid malignancy with a sensitivity of 0.61 and specificity of 0.97 (Figure 1).

### 3.2. Results of Patients in Bethesda DCs III, IV, and V (n = 221)

Due to the inconclusive results found in the literature, the patients in DCs III, IV, and V were separated out, and analysis of the incidence of malignancy in this subgroup was performed. Among the 521 patients, a group of 221 patients who were referred for surgery due to Bethesda DCs III, IV, and V were identified. On the basis of the histopathological examination, two groups were distinguished—malignant and benign—in order to present the results according to the Bethesda category. DC II is included in the table because the logistic regressions for DCs III, IV, and V were calculated for DC II (Table 5).

It was found that patients in DC V were statistically significantly younger than patients in DCs II, III, and IV (*p* < 0.0001). The patients in DC V were, on average, 10 years younger compared to the patients in DCs II, III, and IV.

As there was a statistically significant correlation between the HP results and the cytology results in the patients in DCs II, III, IV, and V, the authors assessed in which groups malignant lesions were more frequent. They were found to be statistically significantly more frequent in DC V compared to DC II (*p* < 0.0001), DC III (*p* < 0.0001), and DC IV (*p* < 0.0001) (Table 6).

Of the 61 patients with histopathologically confirmed lymphocytic thyroiditis (LT), 42.6% were in category V, 13.1% were in category IV, 16.4% were in category III, and 21.9% were in category II. The most commonly observed type of cancer was PTC, with 44 cases (93.0%). In the group of patients with background-confirmed LT, category V was statistically significantly more frequent than category III (*p* = 0.0008), category IV (*p* = 0.0009), and category II (*p* < 0.0001) (Table 7).

The multivariate logistic regression analysis was used to determine the risk of thyroid malignancy in patients in DCs III, IV, and V. The above cytology groups were compared with DC II. The effects of age, sex, cytology score, obesity (BMI ≥ 30.0), and the presence of lymphocytic thyroiditis on the development of thyroid cancer were assessed (Table 8).

Based on the multivariate logistic regression, DC V (*p* < 0.0001) and age (*p* = 0.006) were found to have statistically significant effects on the occurrence of thyroid cancer. In contrast, no statistically significant effects were found to be exerted by DCs III and IV in the presence of a malignant lesion.

**Figure 2:**
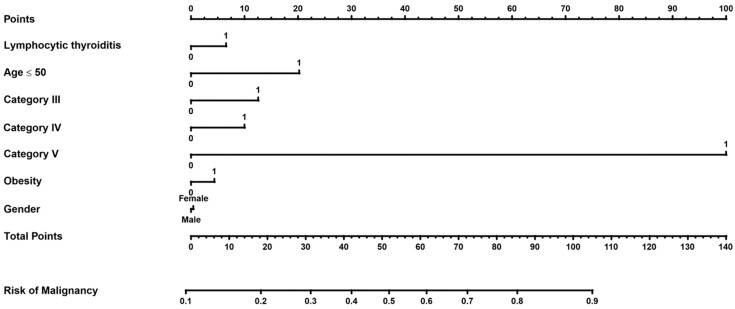
A nomogram to predict the risk of malignancy in the groups in Bethesda categories II, III, IV, and V.

To visualize the results of the multivariate analysis, a nomogram was created to assess the risk of thyroid cancer when several factors were present at the same time. The scores for each parameter were marked on a scale of “points”. The scores were then summed up and displayed on the “total score” scale, which was related to the “risk of cancer” scale (Figure 2).

To demonstrate how to interpret the nomogram, please note that we marked a point on the scales for the parameters in the regression, including gender, LT = 1, age < 50, etc., and then placed the relevant points on the “points” scale. We added up the resulting points and marked them on the “total points” scale, which is related to the “risk of cancer” scale. For example, age < 50 (20 points), sex = male (0 points), DC III = 1 (13 points), DC IV = 0 (0 points), DC V = 0 (0 points), no obesity, i.e., obesity = 0 (0 points), and no lymphocytic Hashimoto’s (0 points). We then added these points, i.e., 20 + 0 + 13 + 0 + 0 + 0 + 0 + 0 = 33, which results in a total points value of 33. When placed on the “risk of cancer” scale, the added value results in a risk of cancer of approximately 30%.

## 4. Discussion

In the study group of 521 patients with thyroid nodules, malignant lesions were found in 197 cases (37.85%), which is similar to the findings from other studies [17]. The vast majority of these lesions were well-differentiated carcinomas (95.4%), which is also consistent with the literature [18]. US-guided FNAB is commonly used as a preoperative diagnostic tool because of its high accuracy [19,20,21]. However, false positive results are common, where the cytology suggests malignancy, but the histopathology does not confirm it. A number of studies have evaluated the diagnostic reliability of biopsies on the basis of histopathological findings [7,22,23,24,25,26]. 

The risk of malignancy (ROM) is important in the assessment of thyroid nodules. The following risk values have been reported for each of the Bethesda DCs I–VI: 1–4%, 0–3%, 5–15%, 15–30%, 60–75%, and 97–99%, respectively [9]. Many investigators have attempted to assess the ROM, with variable outcomes [27,28,29]. In our study, among the 521 patients, different rates of malignant lesions (ROM) were found for the different Bethesda DCs, as follows: I—38.1%, II—15.6%, III—29.8%, IV—18.6%, V—91.0%, and VI—93.2% (Table 9).

It should be emphasized that non-diagnostic FNAB (DC I) can involve any category. In our study, there were false negative category I cytology results in eight cases, where malignancy was confirmed by histopathology. 

In 180 patients in DC II, 28 malignant lesions (15.5%) were found. Similar results have been reported in the literature [30]. In our study, the false negative results for DC II may have been due to the fact that 11 cases were papillary microcarcinomas, which may not have been detected due to their small size. The most common reason for referral to surgery was tracheal compression due to multinodular goiter. In the case of the histopathological confirmation of medullary carcinoma despite being in Bethesda cytology category II, the patients were referred to a surgery department because of elevated calcitonin levels.

For DC III, it is important to add the subcategories AUS, FLUS, and FLUS/AUS [12,31], as AUS is associated with a higher risk of malignancy than the FLUS subcategory and refers mainly to smears with cellular features suggestive of papillary carcinoma. Several studies have assessed the risk of malignancy in the Bethesda III subcategories [32,33,34]. In our study, of the DC III patients, there were 25 thyroid carcinomas (29.7%), and benign lesions were detected in 59 cases (70.2%). In the cytological biopsy results for DC III in our study, we did not have a specific subcategory for the AUS or FLUS type, which is undoubtedly a drawback of our research.

In our study, out of 70 cases in DC IV, only 13 (18.5%) thyroid carcinomas were found, and benign lesions were present in 57 cases (81.5%). The incidence of malignant lesions in this category was estimated to be approximately 37–39% [35,36]. Based on the multivariate regression analysis, the present authors found no statistically significant effect of DCs III and IV on the risk of malignancy. Therefore, with this diagnosis, the risk of malignancy should be assessed together with clinical and ultrasound risk before deciding whether to refer the patient for surgery. 

In our group of patients in DC V, a malignant lesion was found in 91.0% of the subjects, and other lesions accounted for 9%. On the basis of the multivariate regression analysis, this category was found to be statistically significantly associated with the risk of malignancy (*p* < 0.0001, OR-62.34); the ROC curve also showed that from this category onward, the risk of malignancy increased with a specificity of 0.97 and sensitivity of 0.61. Thus, in the case of category V, surgical treatment is indicated.

It should also be noted that 7 of the 40 patients who did not undergo FNAB prior to surgery were also found to have a malignant tumor (PTC) upon HP examination. The lack of FNAB was because the indication for surgery was compression symptoms and thyroid dysfunction, mainly hyperthyroidism. 

Based on our study, it can be concluded that several factors affect the diagnostic performance of thyroid biopsy. The sensitivity was affected by the datasets assigned to the numerator or denominator. The sensitivity increased when more Bethesda categories were included in the numerator. In our study, the sensitivities for categories 2, 3, 4, 5, and 6 were 0.96, 0.81, 0.68, 0.61, and 0.29, respectively. A similar trend was found by Ha et al., who showed, in a study of 4822 thyroid nodules, that sensitivity increased when more Bethesda categories were included in the numerator and when non-diagnostic results were excluded [37].

The PPVs for criterion 5 (categories VI and VI), criterion 4 (categories VI, V, and IV), and criterion 3 (categories VI, V, IV, and III) were 0.92, 0.66, and 0.55, respectively. Based on the ROC curve, criterion 5 (i.e., Bethesda category V and VI tumors) was associated with the presence of thyroid cancer with a specificity of 0.97 and a sensitivity of 0.61. Criterion 5 represents the cut-off point associated with a higher risk of malignancy. 

Our study also analyzed whether demographics, age, gender, and BMI had an effect on the risk of thyroid malignancy. We found that age statistically significantly differed (*p* < 0.0001) between the patients with and without malignancy. The patients with a malignant neoplasm were, on average, eight years younger (mean age (SD) of 47.4 (14.9)) than those diagnosed with a benign lesion (mean age (SD) of 55.3 (14.7)). However, there were no statistically significant differences in gender, BMI, or obesity. A study by Kwong et al. found that the incidence of clinically significant thyroid nodules increases with age, while the risk that such nodules are malignant decreases [38]. Velsen et al. found that the risk that a nodule was cancerous decreased with age [39]. In contrast, Raparia et al. showed that there were no statistically significant differences in age between the benign and malignant lesion groups [40]. 

This study also sought to determine whether lymphocytic thyroiditis (LT) is a risk factor for malignancy. Of our 61 patients with a background of confirmed lymphocytic thyroiditis, 42.6% were in category V, 13.1% were in category IV, 16.4% were in category III, and 21.9% were in category II, which is statistically significant. Some studies have confirmed that differentiated thyroid cancer can coexist with lymphocytic thyroiditis [41], mainly papillary carcinoma [42].

To assess whether cumulative factors affect the risk of malignancy, logistic regression was performed, which included five parameters, i.e., age, sex, BMI, the presence or absence of LT, and Bethesda DCs III, IV, and V. Based on the multivariate regression analysis, DC V and an age ≤ 50 years were statistically significantly associated with the risk of malignancy (*p* < 0.0001 and *p* < 0.006, respectively). 

Other factors influencing the development and course of thyroid cancer are currently being analyzed. Undoubtedly, the role of studies on the influence of miRNAs with unbalanced expression profiles in the development of thyroid cancer should be emphasized. A study by Orlandella et al. showed that deregulation of the miR-331-5p/BID axis can increase the aggressiveness of thyroid cancer cell lines [43]. The authors suggested a potential role of these component factors as biomarkers in thyroid cancer tissues. 

It should be noted that the limitations of our study include its retrospective nature, the single institution design, the lack of EUTIRADS assessment in a significant number of patients prior to surgery, and the lack of fine-needle biopsy in a proportion of patients referred for surgery due to multinodular goiter and compression symptoms. The combined EUTIRADS and Bethesda assessment of the focal lesion would have significantly affected the decision to operate and the extent of surgery.

The large size of the study group was undoubtedly an advantage of our study.

## 5. Conclusions

Based on this study, it can be concluded that in DCs III and IV, consideration may be given to keeping the patient under close clinical, ultrasound, and cytological surveillance without a premature referral for surgery. In the case of DC V, it is advisable to refer the patient for surgery. In our study, we found that patients under 50 years of age had a higher risk of malignancy than older patients. 

In addition, if lymphocytic thyroiditis and a focal lesion coexist, a biopsy should be taken into consideration.

Further research and the development of more reliable diagnostic methods are needed to optimize the management of thyroid nodules and avoid unnecessary surgery.

## Figures and Tables

**Table 1:** Demographic and pathologic features of 521 patients who underwent surgery due to thyroid nodules.

	Total (N = 521)	Benign (N = 324)	Cancerous (N = 197)	*p*-Value
**Sex**				0.94
Female	425 (81.6%)	264 (81.5%)	161 (81.7%)	
Male	96 (18.4%)	60 (18.5%)	36 (18.3%)	
**BMI**				0.52
Mean (SD)	27.3 (4.87)	27.4 (4.76)	27.1 (5.08)	
Median [Q1–Q3]	26.6 [23.5–30.5]	26.7 [23.8–30.5]	26.4 [23.2–30.8]	
Missing	98 (18.8%)	57 (17.6%)	41 (20.8%)	
**Obesity** (BMI ≥ 30)				0.76
No	302 (58.0%)	192 (59.3%)	110 (55.8%)	
Yes	121 (23.2%)	75 (23.1%)	46 (23.4%)	
Missing	98 (18.8%)	57 (17.6%)	41 (20.8%)	
**Age**				<0.0001
Mean (SD)	52.3 (15.2)	55.3 (14.7)	47.4 (14.9)	
Median [Q1–Q3]	53.0 [40.0–65.0]	57.0 [43.8–67.0]	45.0 [36.0–59.0]	
**Cytology**				<0.0001
I	21 (4.0%)	13 (4.0%)	8 (4.1%)	
II	180 (34.5%)	152 (46.9%)	28 (14.2%)	
III	84 (16.1%)	59 (18.2%)	25 (12.7%)	
IV	70 (13.4%)	57 (17.6%)	13 (6.6%)	
V	67 (12.9%)	6 (1.9%)	61 (31.0%)	
VI	59 (11.3%)	4 (1.2%)	55 (27.9%)	
Not performed	40 (7.7%)	33 (10.2%)	7 (3.6%)	
**Lymphocytic thyroiditis**				<0.0001
No	442 (84.8%)	292 (90.1%)	150 (76.1%)	
Yes	79 (15.2%)	32 (9.9%)	47 (23.9%)	
**Malignancy**				
PTC	172 (33.0%)		172 (87.3%)	
FTC	16 (3.1%)		16 (8.1%)	
MTC	5 (1.0%)		5 (2.5%)	
ATC	1 (0.2%)		1 (0.5%)	
OTC	2 (0.4%)		2 (1.0%)	
Lymphoma	1 (0.2%)		1 (0.5%)	
**Thyroid operation**				
Total thyroidectomy	480 (92.1%)	295 (91%)	185 (94%)	
Unilateral lobectomy	30 (5.7%)	22 (6.8%)	8 (4.1%)	
Other operation on thyroid gland	11 (2.1%)	7 (2.2%)	4 (2.0%)	
**Lymph nodes**				
Bilateral central lymph node dissection	191 (36.7%)	89 (27.5%)	102 (51.8%)	
Central lymph node dissection and one-sided lateral lymph node dissection	18 (3.5%)	2 (0.6%)	16 (8.1%)	
Unilateral central lymph node dissection	80 (15.4%)	40 (12.3%)	40 (20.3%)	
One-sided lateral lymph node dissection	7 (1.3%)	2 (0.6%)	5 (2.5%)	
Bilateral lateral lymph node dissection	2 (0.4%)	0 (0%)	2 (1%)	
None	223 (42.8%)	191 (59.0%)	32 (16.2%)	

Legend: Cytology: I—non-diagnostic or unsatisfactory; II—benign; III—atypia of undetermined significance (AUS) or follicular lesion of undetermined significance (FLUS); IV—follicular neoplasm or suspicious follicular neoplasm; V—suspicious for malignancy; VI—malignant; PTC, papillary thyroid carcinoma; FTC, follicular thyroid carcinoma; MTC, medullary thyroid carcinoma; OTC, oncocytic thyroid carcinoma; ATC, anaplastic thyroid carcinoma.

**Table 2:** Comparison of the cytology results with the histopathology results for DCs I–VI.

Cytology	Total, n (%)	Histopathology			
		Benign, n (%)		Cancer, n (%)	
**I**	21 (4.0%)	FA	2 (15.4%)	FTC	2 (25%)
		GD	1 (7.7%)	PTC	6 (75%)
		NG	9 (69.2%)		
		Other diagnosis	1 (7.7%)		
**II**	180 (34.5%)	FA	15 (9.9%)	FTC	4 (14.3%)
		FT-UMP	1 (0.7%)	MTC	3 (10.7%)
		GD	1 (0.7%)	PTC	21 (75%)
		OA	1 (0.7%)		
		LT	1 (0.7%)		
		NG	130 (85.5%)		
		NIFTP	2 (1.3%)		
**III**	84 (16.1%)	FA	14 (23.7%)	FTC	7 (28.0%)
		FT-UMP	2 (3.4%)	OTC	1 (4.0%)
		OA	1 (1.7%)	PTC	17 (68.0%)
		LT	3 (5.1%)		
		NG	35 (59.3%)		
		NIFTP	2 (3.4%)		
		WDT-UMP	2 (3.4%)		
**IV**	70 (13.4%)	FA	15 (26.3%)	OTC	1 (7.7%)
		FT-UMP	5 (8.8%)	PTC	12 (92.3%)
		OA	13 (22.8%)		
		LT	2 (3.5%)		
		NG	18 (31.6%)		
		NIFTP	2 (3.5%)		
		WDT-UMP	2 (3.5%)		
**V**	67 (12.9%)	FA	1 (16.7%)	ATC	1 (1.6%)
		NG	3 (50.0%)	FTC	2 (3.3%)
		NIFTP	1 (16.7%)	PTC	58 (95.1%)
		Other diagnosis	1 (16.7%)		
**VI**	59 (11.3%)	NG	2 (50.0%)	FTC	1 (1.8%)
		Other diagnosis	2 (50.0%)	Lymphoma	1 (1.8%)
				MTC	2 (3.6%)
				PTC	51 (92.7%)
**Not** **performed**	40 (7.7%)	FA	4 (12.1%)	PTC	7 (100%)
		GD	3 (9.1%)		
		OA	2 (6.1%)		
		LT	1 (3.0%)		
		NG	19 (57.6%)		
		NIFTP	3 (9.1%)		

Legend: FA, follicular thyroid adenoma; GD, Graves’ disease; NG, nodular goiter; FTUMP, follicular tumor with uncertain malignant potential; OA, oncocytic adenoma of the thyroid; LT, lymphocytic thyroiditis (Hashimoto’s); WDT-UMP, well-differentiated tumor of uncertain malignant potential; NIFTP, non-invasive follicular thyroid neoplasm with papillary-like nuclear features; FT-UMP, follicular tumor with uncertain malignant potential; PTC, papillary thyroid cancer; FTC, follicular thyroid cancer; MTC, medullary thyroid cancer; ATC, anaplastic thyroid cancer; OTC, oncocytic thyroid carcinoma.

**Table 5:** Characteristics of patients in DCs II, III, IV, and V included in multivariate logistic regression.

	Total (N = 401)	II (N = 180)	III (N = 84)	IV (N = 70)	V (N = 67)	*p*-Value
**Sex**						0.39
Female	326 (81.3%)	140 (77.8%)	69 (82.1%)	60 (85.7%)	57 (85.1%)	
Male	75 (18.7%)	40 (22.2%)	15 (17.9%)	10 (14.3%)	10 (14.9%)	
**Age**						0.00001
Mean (SD)	53.6 (14.7)	55.2 (14.4)	54.6 (12.0)	55.8 (16.3)	45.8 (14.3)	
Median [Q1–Q3]	54.0 [42.0–66.0]	56.0 [44.0–67.0]	57.0 [44.0–65.3]	58.0 [42.0–68.0]	42.0 [35.5–56.0]	
**Histological main diagnosis**						<0.0001
Benign	274 (68.3%)	152 (84.4%)	59 (70.2%)	57 (81.4%)	6 (9.0%)	
Cancerous	127 (31.7%)	28 (15.6%)	25 (29.8%)	13 (18.6%)	61 (91.0%)	
**Lymphocytic thyroiditis**						<0.0001
No	340 (84.8%)	163 (90.6%)	74 (88.1%)	62 (88.6%)	41 (61.2%)	
Yes	61 (15.2%)	17 (9.4%)	10 (11.9%)	8 (11.4%)	26 (38.8%)	

**Table 6:** Comparison of the presence of malignant lesions among patients in DCs II, III, IV, and V.

Bethesda Categories	Malignancy	
	n (%)/n (%)	*p*-Value
III vs. II	25 (29.8%)/28 (15.6%)	0.019
III vs. IV	25 (29.8%)/13 (18.6%)	0.161
III vs. V	25 (29.8%)/61 (91.0%)	<0.0001
II vs. IV	28 (15.6%)/13 (18.6%)	0.572
II vs. V	28 (15.6%)/61 (91.0%)	<0.0001
IV vs. V	13 (18.6%)/61 (91.0%)	<0.0001

**Table 7:** Frequency of Bethesda categories II, III, IV, and V among patients with a background of confirmed lymphocytic thyroiditis.

Bethesda Categories	Lymphocytic Thyroiditis	
	n (%)/n (%)	*p*-Value
III vs. II	10 (11.9%)/ 17(9.4%)	0.98
III vs. IV	10 (11.9%)/ 8(11.4%)	0.99
III vs. V	10 (11.9%)/ 26(38.8%)	0.0008
II vs. IV	17 (9.4%)/ 8(11.4%)	0.98
II vs. V	17 (9.4%)/ 26(38.8%)	<0.0001
IV vs. V	8 (11.4%)/ 26(38.8%)	0.0009

**Table 8:** Multivariate logistic regression analysis for Bethesda categories II, III, IV, and V.

Variable	OR per	Univariate		Multivariate	
		OR (95% CI)	*p*-Value	OR (95% CI)	*p*-Value
Sex	Female/male	0.87 (0.51–1.51)	0.63	1.02 (0.47–2.18)	0.96
Lymphocytic thyroiditis	Yes/no	2.85 (1.63–4.96)	0.0002	1.31 (0.55–3.11)	0.54
Bethesda III	Yes/no	0.89 (0.53–1.51)	0.67	1.68 (0.81–3.47)	0.16
Bethesda IV	Yes/no	0.43 (0.23–0.83)	0.011	1.51 (0.69–3.32)	0.30
Bethesda V	Yes/no	41.28 (17.11–99.61)	<0.0001	62.34 (20.16–192.8)	<0.0001
Age ≤ 50	Yes/no	2.67 (1.74–4.12)	<0.0001	2.31 (1.27–4.19)	0.006
Obesity (BMI ≥ 30)	Yes/no	0.90 (0.53–1.51)	0.68	1.2 (0.62–2.31)	0.59

**Table 9:** Comparison of the risk of a neoplasm detected in our study with the results of other studies [9,11,27,28,29].

Bethesda Category	TBSRTC [9], First Edition	TBSRTC [11], Second Edition	ROM of Our Study, n = 521	ROM of Inabnet et al. [27], n = 1746	ROM of Anand et al. [28], n = 646	ROM of Zarif at al. [29], n = 373
I—Non-diagnostic	1–4%	5–10%	38.8%	19.2%		34.6%
II—Benign	0–3%	0–3%	15.6%	12.7%	8.5%	15.6%
III—AUS/FLUS	5–15%	10–30%	29.8%	31.9%	66.7%	50%
IV—FN/SFN	15–30%	25–40%	18.6%	31.4%	63.6%	52%
V—SFM	60–75%	50–75%	91.0%	77.8%	100%	95.7%
VI—Malignant	97–99%	97–99%	93.2%	96.0%	100%	100%

## Data Availability

The original contributions presented in this study are included in this article. Further inquiries can be directed to the corresponding authors.
